# Liquid condensates come of age: Nanoscale domains at the crossroads of stress and disease

**DOI:** 10.1002/ctm2.70833

**Published:** 2026-09-11

**Authors:** Nils G. Walter, Bisal Halder

**Affiliations:** ^1^ Center for RNA Biomedicine, Single Molecule Analysis Group, Department of Chemistry University of Michigan Ann Arbor Michigan USA

## 1 FROM CELLULAR COMPARTMENTS TO DYNAMIC CONDENSATES

**FIGURE 1:**
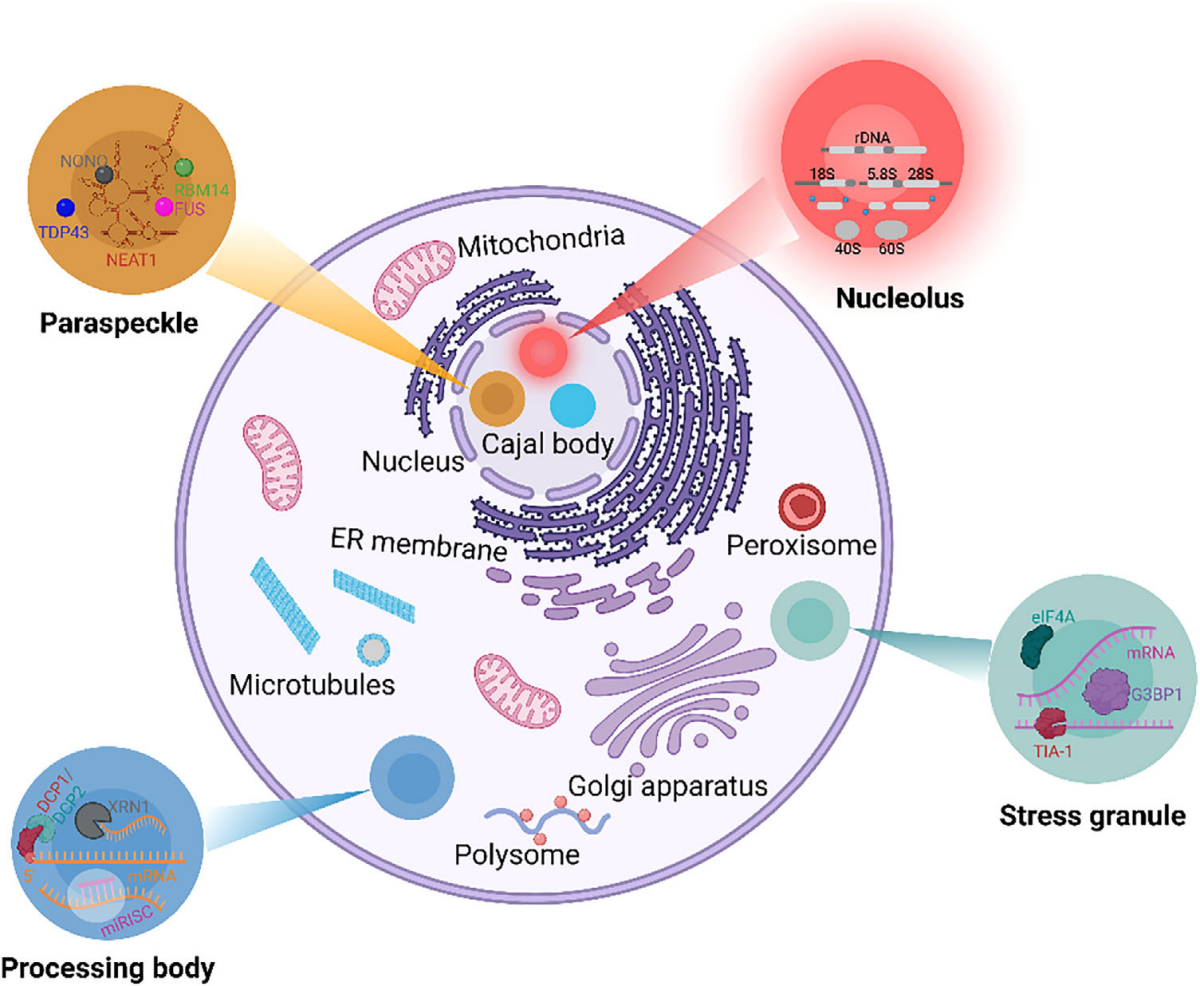
Intracellular organisation from membrane‐bound organelles to dynamic biomolecular condensates: Schematic overview of eukaryotic cell compartmentalisation. Classical membrane‐bound organelles (mitochondria, endoplasmic reticulum, Golgi apparatus, peroxisomes) provide physical boundary membranes. In contrast, membrane‐less organelles (MLOs) form via multivalent protein–RNA interactions to dynamically organise specific cellular chemistry. Insets highlight key nuclear MLOs—including the paraspeckle (scaffolded by lncRNA *NEAT1* with host proteins FUS, TDP‐43, NONO and RBM14 to regulate gene expression via nuclear RNA retention) and the nucleolus (which coordinates ribosomal DNA transcription and ribosome subunit assembly)—alongside cytoplasmic MLOs, including stress granules (which temporarily sequester stalled mRNAs and proteins involved in translational regulation, such as TIA‐1 and eIF4A, with key stress‐granule nucleator G3BP1 to protect them during cellular stress) and processing bodies (P‐bodies, which act as dedicated hubs for mRNA decay, storage and gene silencing and house, e.g., DCP1/DCP2, XRN1 and miRISC).

Human cells organise their contents into distinct compartments to optimise cellular function. These include classical membrane‐bound organelles—such as the nucleus, endoplasmic reticulum, endosomes and mitochondria (Figure 1)—whose architecture and composition are dynamically regulated through processes including remodelling, fission, fusion and vesicular trafficking; as well as membrane‐less organelles (MLOs) and biomolecular condensates that reversibly assemble through multivalent protein–protein, protein–RNA and RNA–RNA interactions. Examples for MLOs include the nucleolus, stress granules and processing bodies (Figure 1). Unlike membrane‐bound compartments, condensates can rapidly alter their composition and material state as local concentrations and molecular interactions change, potentially within seconds to minutes, thereby enabling cellular organisation to adapt dynamically to changing physiological demands and stresses.^1^

This dynamism may be especially important for RNA–protein assemblies, where multivalent RNAs often scaffold and concentrate specific proteins, promoting MLO formation without the need to form or dissolve membrane‐bound compartments.^2^ Indeed, a major conceptual advance in our understanding of biomolecular condensation emerged from observations that structures such as nematode *Caenorhabditis elegans* P granules behave as liquid‐like phases, undergoing condensation, fusion and dissolution in response to cellular cues.^3^

## 2 FROM LIQUID DROPLETS TO STRUCTURED MATERIALS

Biomolecular condensates are often depicted as homogeneous liquid droplets. In reality, their material states can span a continuum from highly dynamic liquids to viscoelastic networks, gels and, in some cases, solid or fibrillar assemblies.^4^ Under certain conditions, initially dynamic condensates can progressively ‘age’, developing increasingly long‐lived intermolecular contacts and—in some systems—percolated polymer networks, with more dynamic exchange limited to their surfaces. Such spatial and temporal heterogeneity can generate a layered, or core–shell, organisation in which molecules at the periphery remain more exchangeable, whereas those incorporated into a more strongly interlinked interior become increasingly sequestered.

Their properties enable condensates to interrogate and recruit molecules from their surroundings, contributing to storage, degradation or locally accelerated biochemical reactions. Molecules that transition from a dynamic shell into a more constrained interior may thereby become committed to longer residence times, while active cellular processes, including ATP‐dependent remodeling and motor‐driven extrusion, can in some contexts return trapped components to a more mobile state.

This emerging view challenges a simple liquid‐droplet model in which all molecules within a condensate experience essentially equivalent environments. Indeed, compositionally distinct sub‐compartments and non‐equilibrium material states are increasingly recognised as fundamental features of biomolecular condensates rather than exceptions to an otherwise homogeneous phase.^4^, ^5^

**FIGURE 2:**
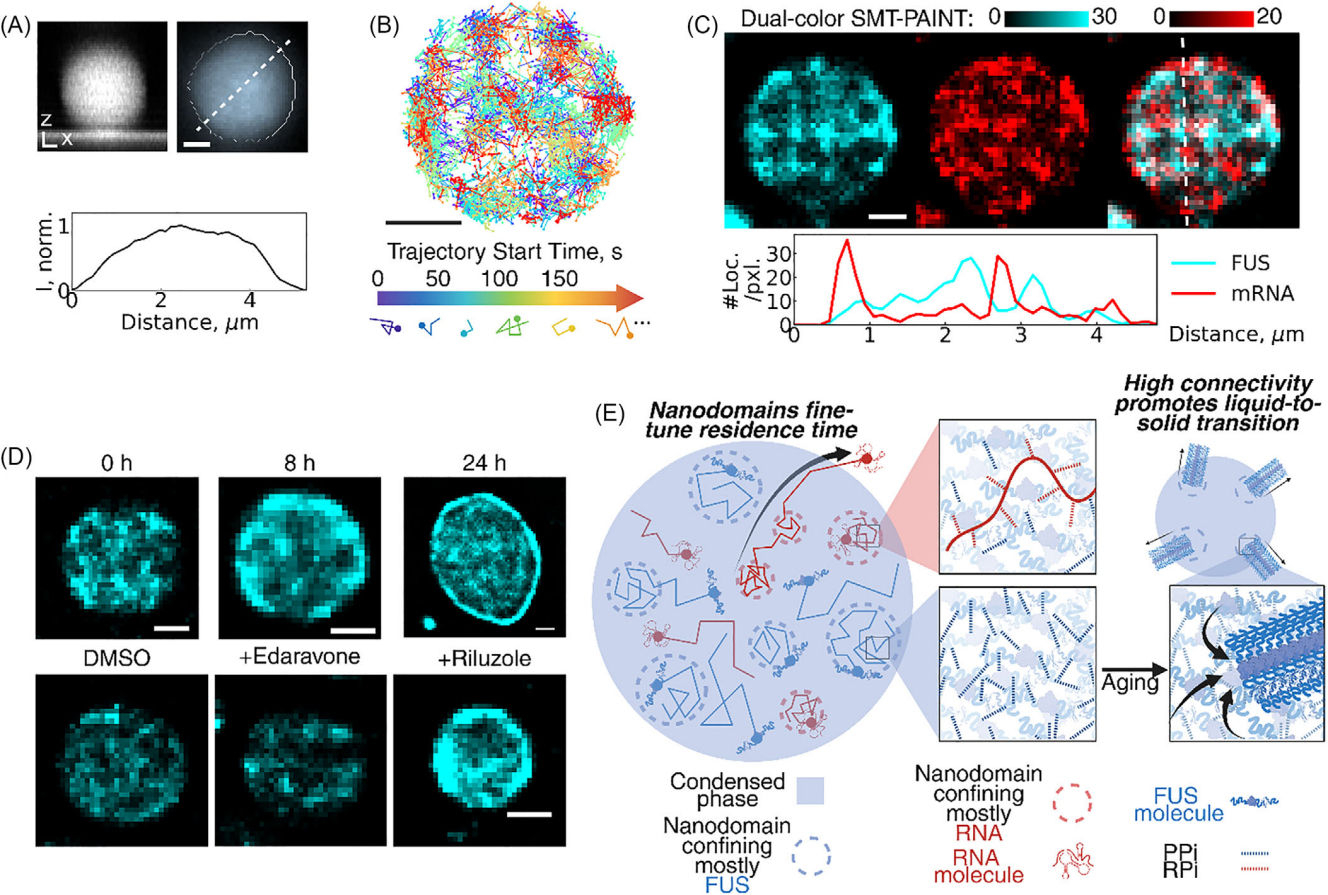
Nanoscale domain architecture, material ageing and small‐molecule remodelling in fused‐in‐sarcoma (FUS) condensates. (a) Confocal cross‐section and intensity profile of a representative FUS protein droplet, illustrating macroscopic liquid‐like homogeneity at conventional resolutions. (b) Single‐molecule tracking (SMT) trajectories colour‐coded by time (0–150 s), capturing dynamic molecular diffusion paths inside the condensate. (c) Dual‐colour super‐resolution reconstruction and cross‐sectional density heatmap profiles demonstrating that host protein FUS (cyan) and guest mRNAs (red) segregate into discrete nanodomains. (d) Time‐course fluorescence images showing progressive migration of nanodomains towards the outer boundary over 24 h (top row) to form a gel‐like shell, with this structural reorganisation accelerated by (bottom row) FDA‐approved ALS drugs edaravone and riluzole relative to the control (+DMSO, or dimethyl sulfoxide). (e) Schematic model connecting nanoscale organisation with condensate ageing. Internal nanodomains locally confine protein and RNA diffusion, extending molecular residence times. Over time, nanodomain migration to the surface and increased polymer connectivity converts the periphery into a dense shell, driving a liquid‐to‐solid transition and potentially nucleating surface‐associated protein fibrils. Adapted with permission from Springer Nature: Nature Nanotechnology.^6^

In a recent single‐molecule tracking study, we discovered a second mode of transition between more dynamic and more sequestered molecular behaviours: the capture of individual molecules within approximately 100–200‐nm‐scale nanodomains embedded within otherwise liquid‐like biopolymer droplets (Figure 2).^6^ These nanodomains likely emerge through local percolation during condensate ageing without an accompanying increase in local molecular density (Figure 2a). Distinct nanodomains formed by fused‐in‐sarcoma (FUS) protein host and mRNA guest molecules locally confine their diffusion (Figure 2b,c). As a consequence, molecules within these percolated domains exhibit prolonged residence timeswithin the condensate.

Over time, the nanodomains tend to relocate towards the condensate surface, potentially driven by surface tension, differences in interfacial chemistry or other energetic and kinetic factors (Figure 2d). This reorganisation converts the shell into an increasingly rigid, gel‐like material. Eventually, organised protein fibres often grow from the condensate surface, providing a potential mechanistic connection between nanoscale condensate organisation and the pathological fibrillar assemblies associated with neurodegenerative disease (Figure 2e).^6^

This nanoscale organisation therefore adds a regulatory layer between molecular interactions and mesoscale phase separation. Rather than viewing a condensate simply as a liquid compartment with a defined composition, it may be more appropriate to view it as a dynamic material whose internal architecture determines molecular residence times, exchange pathways and ultimately the biological fates of its components.

## 3 SMALL MOLECULES CAN REMODEL CONDENSATE ARCHITECTURE

Intriguingly, small‐molecule compounds, including the FDA‐approved amyotrophic lateral sclerosis (ALS) therapeutics edaravone and riluzole, accelerate the reorganisation of protein nanodomains towards the condensate shell (Figure 2d).^6^ This observation raises the possibility that membrane‐permeable small molecules more generally can alter the internal architecture, material properties and ageing trajectories of biomolecular condensates.

This possibility offers important therapeutic opportunities. High‐throughput screening approaches increasingly seek compounds that modulate phase separation in cells, with the goal of manipulating pathological protein condensation and aggregation.^7^ Yet if the most toxic species in neurodegenerative disease are small oligomeric assemblies or other transient intermediates rather than the larger, apparently inert—even neuroprotective—fibrils, simply promoting or preventing fibril formation may not necessarily be therapeutically desirable.^8^ More subtle, mechanistically informed strategies may be required—strategies that target molecular exchange, nanodomain organisation or condensate ageing trajectories, rather than phase separation per se.

The observation that approved drugs can alter condensate architecture also raises an intriguing translational possibility: the material state of a condensate could become a previously underappreciated pharmacological target. Rather than asking only whether a compound increases or decreases condensation, future screens might ask whether it changes where molecules reside within a condensate, how long they remain there and how their behaviour in the condensate evolves over time.

## 4 STRESS GRANULES: ONE NAME, MANY CONDENSATES?

We still lack a complete picture of the condensation reactions that occur throughout the cell. This is particularly evident for stress granules (Figure 1). Classically, stress granules are induced experimentally by severe oxidative stress, often through addition of sodium arsenite to cultured cells—a condition that arguably resembles chemical poisoning in an Agatha Christie novel more than a physiologically relevant challenge. Such perturbations robustly induce large RNA‒protein assemblies, but they may not faithfully represent the diverse condensates generated by physiological stresses. Different stressors, cell types and cellular states can produce assemblies with distinct compositions, dynamics and material properties.^9^

Thus, rather than a single universal ‘stress granule’, the cell may deploy a plethora of distinct stress‐responsive RNA–protein condensates, each tuned to a particular perturbation and cellular context. Such assemblies could function as rapid responders to many distinct types of stresses: by concentrating, releasing or temporarily sequestering selected RNAs and proteins, they could reconfigure gene expression and RNA metabolism with high molecular precision on timescales far faster than transcriptional reprogramming or membrane‐bound organelle biogenesis.

This possibility suggests a broader conceptual framework in which RNA–protein condensates constitute an adaptive stress‐ and environmental cue‐response layer of cellular organisation. Stress could trigger rapid condensation; condensation could generate nanoscale organisation; nanoscale organisation could alter molecular residence times and reaction kinetics; and prolonged stress or altered homeostasis could drive remodelling and ageing towards pathological material states. The transition from adaptive to maladaptive condensation may therefore depend not simply on whether a condensate forms, but on how its internal architecture and composition evolve with time.

## 5 FROM THE NUCLEOLUS TO THE MODERN CONDENSATE

The emerging multitude of distinct MLOs is notoriously difficult to study. The nucleolus was formally described nearly two centuries ago, with the first properly documented accounts attributed independently to Rudolf Wagner (1835) and Gabriel Gustav Valentin (1836, 1839).^10^ Yet our mechanistic understanding of the physicochemical principles underlying rapid condensate assembly and disassembly is less than two decades old, dating in large part to the realisation that P granules can behave as liquid‐like phases.^1^, ^3^

Over these last two decades, the field has undergone a remarkable conceptual progression: from organelles as static structures, to condensates as dynamic liquid phases, and now towards condensates as internally organised, evolving materials.^4^ The latter transition may be particularly important for translational medicine because condensate function is likely to depend not only on composition or concentration, but also on molecular mobility, residence time, nanoscale organisation and material history.

Their dynamic nature and rapidly changing properties render condensates refractory to static endpoint measurements, including many current omics approaches: a molecular inventory can tell us what is present, but not necessarily where molecules reside, how long they remain there, how rapidly they exchange, or how those properties change as a condensate responds to stress and ages.

Future advances in single‐molecule probing within increasingly physiological contexts—from reconstituted systems and cell lysates to cultured cells and ultimately living tissues—will be needed to move beyond a molecular inventory towards a mechanistic understanding of how condensates adaptively organise cellular chemistry in physiology, stress response and disease.

## AUTHOR CONTRIBUTIONS

Nils G. Walter drafted the manuscript. Bisal Halder contributed edits to the draft and prepared the figures.

## CONFLICT OF INTEREST STATEMENT

The authors declare no conflicts of interest.
